# Does the death of a child influence parental use of psychotropic medication? A follow-up register study from Finland

**DOI:** 10.1371/journal.pone.0195500

**Published:** 2018-05-02

**Authors:** Mikael Rostila, Netta Mäki, Pekka Martikainen

**Affiliations:** 1 Department of Public Health Sciences, Stockholm University, Stockholm, Sweden; 2 Centre for Health Equity Studies, Stockholm University/Karolinska Institutet, Stockholm, Sweden; 3 City of Helsinki Urban Facts, Helsinki, Finland; 4 Population Research Unit, Faculty of Social Sciences, University of Helsinki, Helsinki, Finland; 5 Max Planck Institute of Demographic Research, Rostock, Germany; University of Manitoba, CANADA

## Abstract

**Background:**

Several studies have found that the loss of a child is associated with psychiatric health problems, yet few studies examined whether child loss influences psychotropic medication use. This study examined short- and long-term use of psychotropic medication, both before and after the death of a child, and its potential effect modifiers.

**Methodology/Principal findings:**

A random sample of 205,456 parents, including 902 bereaved parents, were selected from a Finnish total population registry. The analyses were based on linear regressions using generalised estimation equations (GEE) and adjusted for sociodemographic factors. Annual psychotropic use was defined as having purchased prescribed psychotropic medication between 1996 and2012. Bereaved parents were followed for four years prior to and up to four years after the death of their child. An increase in the use of antidepressants and anxiolytics was found in parents following their loss. The highest percentage of use was found around one year after bereavement, followed by a steady decrease although this remained higher than the level of use among non-bereaved four years after the death. Between 20–25% of bereaved mothers and 10–15% of bereaved fathers used antidepressants or anxiolytics one year after bereavement while the corresponding number in non-bereaved was 5–10%. An increase in psychotropic medication was also found several years before the disease-related loss of a child.

**Conclusions/Significance:**

The use of psychotropic medication is markedly higher among parents after losing a child. Patterns of use leading up to and following the death of a child should be further examined in relation to clinical risk factors so as to identify at risk populations.

**Medical subject headings:**

Bereavement, child death, psychotropic medication, death, child, register, Finland

## Introduction

In high income countries, the loss of a child is often rare and unexpected. Hence, it is considered to be one of the most stressful and traumatic life events a person may encounter, especially when the event occurs early in the child’s and parent’s lives [1,2]. While previous studies have found that the loss of a child has significant consequences for parental mental health in the period immediately following their death [3], few studies have examined how this loss impacts the use of psychotropic medication both before and after the event.

A review by Stroebe et al. suggests that bereavement–such as the death of a spouse or child–is associated with several psychological symptoms and illnesses [3,4]. Individual reactions to bereavement include suicidal ideation [5–7]; insomnia [8]; depression, anxiety and depression [9–11]; somatic symptoms; social dysfunction [12]; psychiatric hospitalization [13] and suicide [14]. These reactions are most likely to arise in the early stages of bereavement [3,15–17]. They may also be strongly related to the use of psychotropic medication.

Previous evidence on bereavement and the consequent use of psychotropic medication is sparse, and to our knowledge few studies have examined child loss and psychotropic medication use specifically, especially prior to bereavement. Lacasse and Cacciatore found that 37.5% of bereaved parents who suffered the perinatal/neonatal death of a child were prescribed medication–primarily antidepressants–to help to deal with their loss [18]. Wall-Wieler et al. found that elevated rates of depression, anxiety and psychotropic medication use after the death of an infant end within 1 year of the child’s death [19]. Other studies have found that the loss of a spouse or partner is associated with an increased use of anti-depressive medication. For instance, Möller et al. showed that prescribed psychotropic medication was more common among widowed individuals than married partners, especially among those recently bereaved [20]. A study on cohabitees of cancer patients also found that exposed individuals were more likely to receive a prescription for antidepressant or hypnotic medication both before and after the death of their spouse or partner [21]. Finally, Oksuzyan et al. found that the average daily use of all-cause and major system-specific medications increased from one year before to five years after their spouse’s death, with no sex-specific patterns in the trajectories of medication use [22]. We thus hypothesizse that the death of child is also associated with increased psychotropic use around the time of death.

Bereaved individuals often experience acute suffering, particularly in the initial stages of loss [10,23,24]. Some research indicates that grief following child loss usually persists for the remainder of a parent’s life [10,25], while other studies support the shorter-term consequences of child bereavement [9,24]. This variability may reflect differences in the way bereavement outcomes are measured. For instance, previous studies have indicated an increased risk of mortality in bereaved mothers and parents in general, particularly with regards to external causes of death (i.e. suicide, homicide or accident) [26]. However, the empirical evidence on whether child bereavement contributes to psychotropic medication use is sparse, so we cannot extrapolate such patterns to the use of psychotropic medication without further investigation.

From a health policy perspective, the study of bereavement and use of psychotropic medication is crucial. Although the use of psychotropic medication may be considered an indicator of depression and mental health problems, it may also paradoxically be seen as a means to avoid more severe long-term outcomes, such as psychiatric illness and suicide following bereavement [13,14], both in general and for child loss in particular. The potential harms of psychotropic medication use in relation to bereavement are also unknown [27]. It is therefore highly important to study the duration of psychotropic medication use following child death.

Our aim is to complement the literature on the health effects of bereavement by studying parents’ use of psychotropic medication (including antidepressants, anxiolytics and antipsychotics) both before and after the death of a child, using nationally representative population registry data from Finland. These data are unique as they are not prone to non-participation bias during study recruitment, loss to follow-up or reporting bias on mental health. We postulate that the effect of a child’s death on the use of psychotropic medication will depend on the time before and after the death, the parent’s sex, the nature of the child’s death (disease or external cause) and the age of the child at the time of death.

## Methods

The 2000 Family Study includes a 20% random sample of Finnish households with at least one child aged 0–14 years at baseline in the end of 2000, with individual-level information on all household members (total n = 415,000) supplemented with a 20% random sample of 0–14-year-olds not living in private households (n = 1,600). In addition, all non-resident biological and adoptive parents (n = 28,000) of the 0–14-year-olds in the two samples were included. The data were drawn from population registers at Statistics Finland, with follow-up on mortality data until the end of 2012, and extensive measurement of sociodemographic factors for the period from 1970 to 2012 on all study subjects. Using unique personal identification numbers, these data were linked with complete prescription register information from the Social Insurance Institution of Finland on medication purchases. The prescription register includes data on the date of purchase and type of medication according to the Anatomical Therapeutic Chemical (ATC) classification system. The register holders have approved the use of their data for scientific research. The study has also been approved by Statistics Finland Board of Ethics (Permission TK-53-525-11). Due to data protection regulations of the administrative sources providing the register data, the authors do not have the permission to make the data available to third parties. Interested researchers have the possibility to obtain data access directly by contacting Statistics Finland (tutkijapalvelut@stat.fi, +358917342758) and the Social Insurance Institution (toimistopalvelut@kela.fi, +358206341364).

For the purposes of this study, we defined children as those who were aged 0–14 years at the end of 2000, regardless of their age and family status at the time of death in the 2001–2011 follow-up period. Altogether, there were 205,456 parents in the sample, including 474 mothers and 428 fathers who lost a child. Biological parents were primarily followed-up for the use of psychotropic medicine, regardless of whether they were living with their children or not. However, in case information on the biological parent was missing (1,557 children), we included the resident adoptive parent in the data. 10 mothers and 13 fathers died during the follow-up, and less than one percent of parents migrated in any given year of follow-up. Those dying were censored at the date of death and those migrating at the end of the year before migration.

There were 209 stepmothers and 764 stepfathers included in the dataset for children who did not have information on their biological mother/father. Of these, four mothers and 20 fathers experienced the death of a child. Adjustment for whether a parent was biological or adoptive had very little effect on the outcomes (*analyses available upon request*). In the few cases that parent lost two children (9 mothers and 9 fathers), we analyzed the death of the youngest child (*sensitivity analyses indicate that excluding these cases had little effect on our results; analyses available upon request*). We also studied the nature of the child’s death and its effect on psychotropic use. We categorized deaths accompanied by the ICD-10 codes A00-R99 and X45 as disease-related, as they cover all diseases as well as accidental poisoning by alcohol. The remainder of the deaths were considered external, including accidents, suicides, assaults and sequelae of assaults, and all other external causes.

Annual psychotropic use was defined as having purchased prescribed psychotropic medication between 1996 and 2012. We followed medication use on the basis of exact day of purchase for four years (i.e. four 12 month periods) before the exact date of death a child, and between one to four years after their death. S1 Fig shows the structure of the data by period, one-year study cohorts, and year. For the comparison group of parents who did not lose a child, a reference date in the period 2001–2012 was randomized and medication use was followed around this date. This study covered the use of psycholeptics and psychoanaleptics (ATC-codes N05 & N06), excluding medication for dementia (N06D). These were further divided into three groups: (1) antidepressants (N06A and N06CA01), but excluding tricyclic medication (N06AA, except N06AA22 and N06AA24) which is commonly used for non-psychiatric indications [28,29]; (2) anxiolytics, sedatives and hypnotics (N05B and N05C); and (3) other psychotropic medication (N05A, N06B, and N06C). In the latter group the vast majority of prescriptions were antipsychotic. These three are the most common classes of psychotropic medication used for the treatment of major depressive disorder [30]. The setting for prescription and purchase of psychotropic medication in Finland has been described in more detail elsewhere [30]. Table 1 shows the proportion of parents with purchases of different types of psychotropic medication.

**Table 1:** Total number of parents and number of parents whose child died and the proportion with purchases of psychotropic medication in 1997–2012.

Total number of parents		Antidepressant	Anxiolytics	Antipsychotics
		medication^1^ (%)	medication^2^ (%)	medication^3^ (%)
Mothers	104 047	9,9	6,5	2,1
Farthers	101 409	5,4	5,1	1,6
*Total*	*205 456*	*7*,*7*	*5*,*8*	*1*,*9*
Number of parents whose child died				
Mothers	474	18,3	14,9	4
Farthers	428	9,6	9,1	2
*Total*	*902*	*14*	*12*,*1*	*3*,*1*

1 ATC-codes N06A and N06CA01 (excluding tricyclic medication N06AA, except N06AA22 and N06AA24).

2 ATC-codes N05B and N05C.

3 ATC-codes N05A, N06B, and N06C.

**Fig 1:**
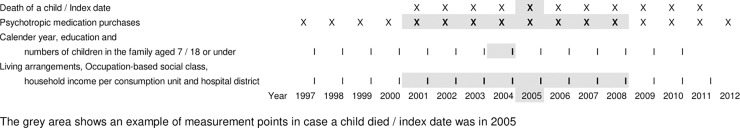
Measurement points for different factors in the study.

We used several socio-demographic factors as control variables and potential effect modifiers. The models were adjusted for age, parental education (basic schooling, secondary and tertiary education) and for the number of children in the family aged 7 or under and 18 and under. These were all measured at the end of the year preceding the death of a child for bereaved parents, or the reference date for parents who did not lose a child. Furthermore, the models were adjusted for parental living arrangements (living alone, married with/without children, single mothers/fathers, cohabiting with children, unknown), occupation-based social class (10 categories), household income per consumption unit and hospital district, all as yearly time-varying variables. Furthermore, the models where mothers and fathers were studied together were adjusted for sex. S1 Table shows the distribution of the covariates adjusted for in the models by whether a parent lost a child or not. Fig 1 shows the measurement points for different variables with an example of a death date or reference date taking place in 2005.

The analyses of psychotropic medication use were based on linear regressions using generalised estimation equations (GEE), with the results shown as the prevalence of use of psychotropic medication. The repeated measures of medication use per subject are highly dependent on each other, and GEE accounts for this interdependence between repeated within-subject measurements by assigning them a correlation structure [31]. An autoregressive correlation structure was chosen on the assumption that the correlation is stronger between observations that are closer to each other in time.

Associations between the death of a child and the prevalence of psychotropic medication use were assessed by examining interactions between parental bereavement status and the study year, i.e. assessing psychotropic medication use in the years before and after the death of child or reference date, and by plotting the model-based estimates of medication use as graphical trajectories.

## Results

**Fig 2:**
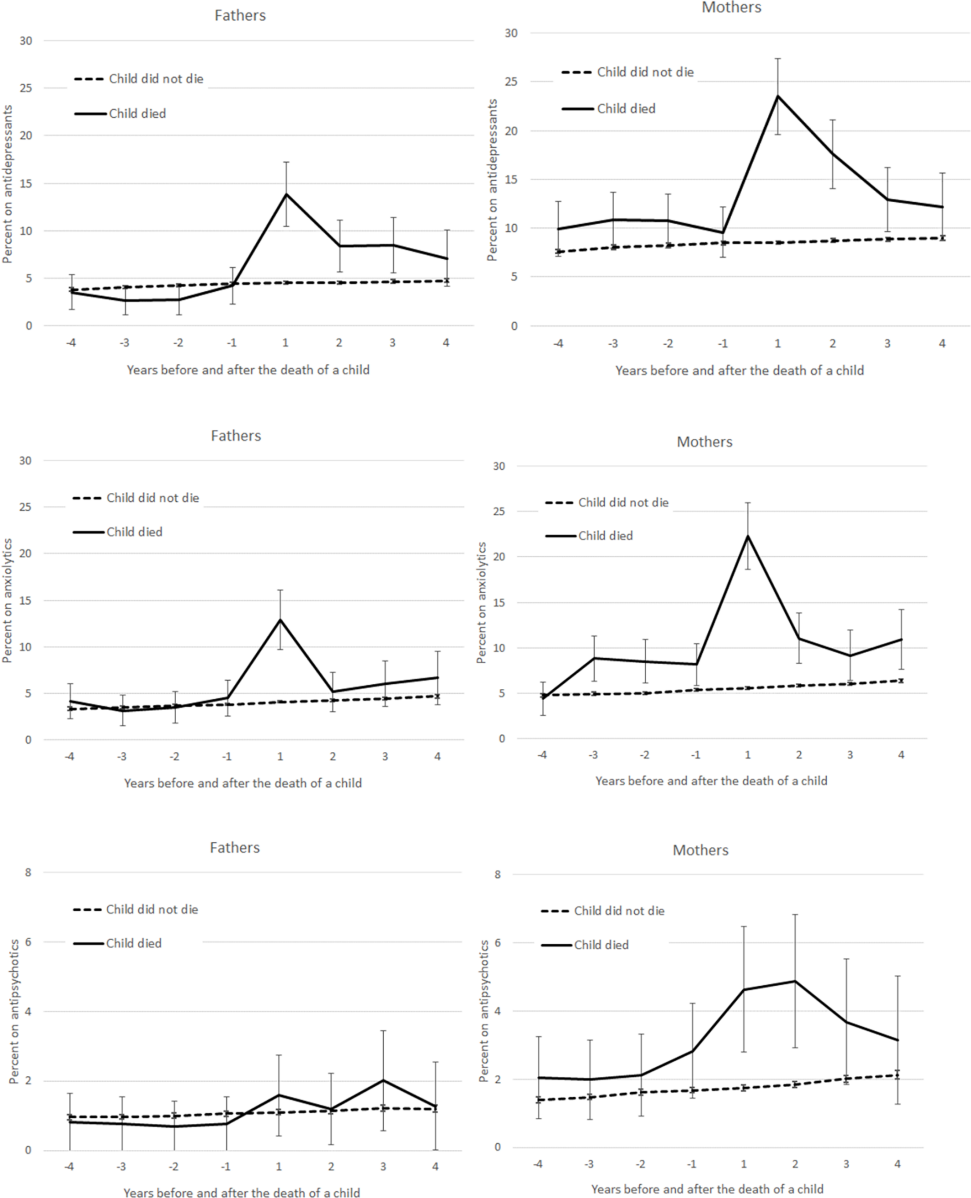
Fathers’ and mothers’ use of psychotropic medication (percent with 95% Cis) four years before and after the death of a child by medication type (antidepressants, anxiolytics and antipsychotics). Adjusted for parental age, calendar year, number of children in the family under the ages of 7–18, health care district, living arrangements, education, occupation based on social class and household income per consumption unit.

We found a marked increase in the use of antidepressants and anxiolytics in mothers and fathers following the death of a child, with the highest percentage of medication use around one year after their child’s death (Fig 2). Even though medication use decreased after this point, it was still higher than among non-bereaved parents four years after the death. Between 20–25% of bereaved mothers and 10–15% of bereaved fathers used antidepressants or anxiolytics one year after bereavement, while the corresponding number in non-bereaved parents was 5–10%. The use of anxiolytics was also slightly increased among mothers several years before the death of a child, but not for fathers.

Furthermore, the use of antipsychotics increased among bereaved mothers in the first two years following the death of their child. While about 2% of the non-bereaved mothers used antipsychotics, the corresponding figure was about 5% among bereaved mothers. There were no differences in the use of antipsychotics among fathers.

**Fig 3:**
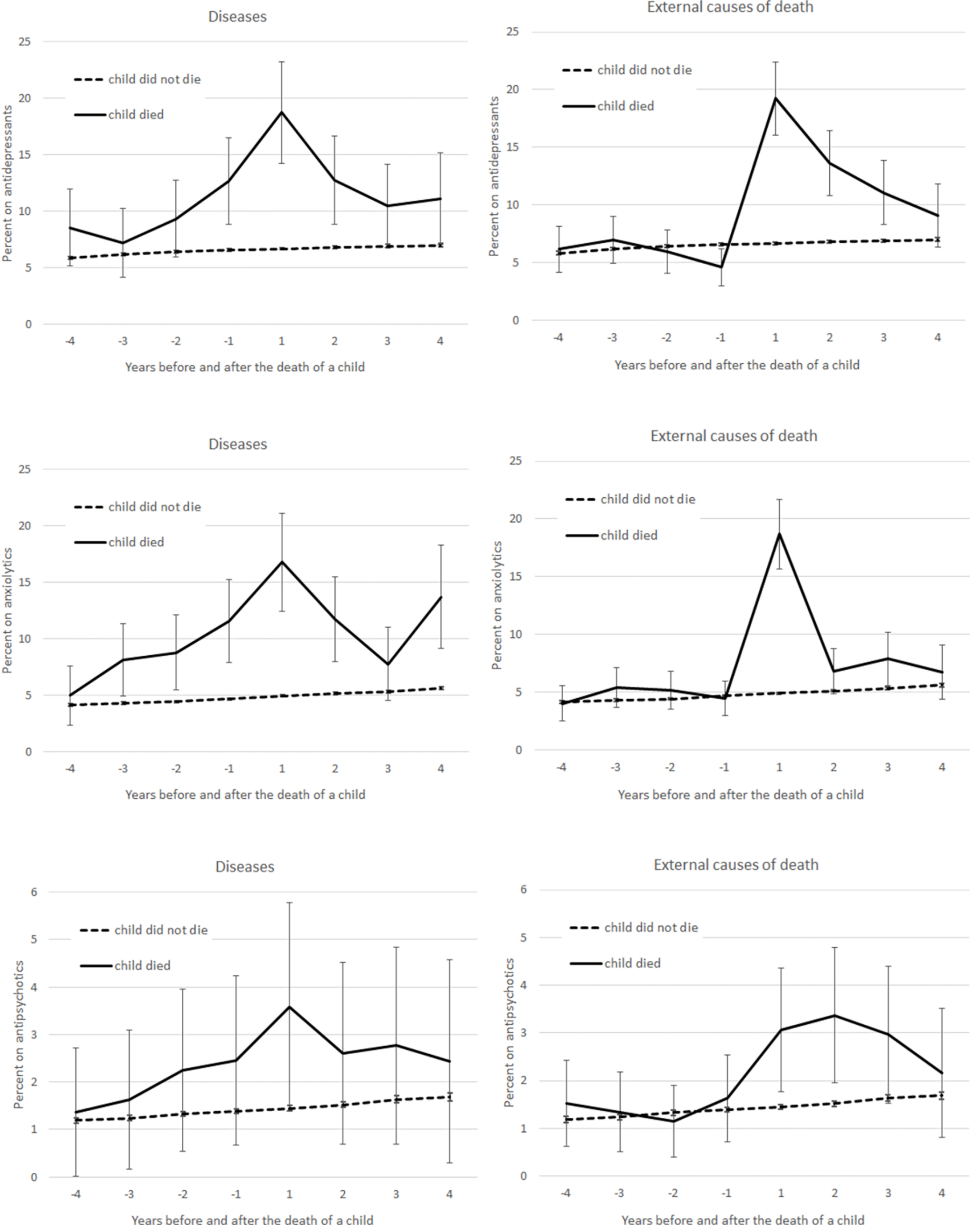
Parental use of psychotropic medication (percent with 95% Cis) four years before and after the death of a child by medication type (antidepressants, anxiolytics and antipsychotics) and cause of death of a child. Adjusted for parental age and sex, calendar year, number of children under the ages of 7 and 18, health care district, living arrangements, education, occupation based on social class and household income per consumption unit.

Although the level of psychotropic medication use differed by sex, the shape of the trajectories were similar. In order to have a sufficient number of cases in the analyses covering the nature of the child’s death, we thus combined the sexes and adjusted for the variable instead (Fig 3). An increase in psychotropic medication use was found both several years before and after disease-related deaths. When the cause of death was external, we found no excess in medication use preceding the death of a child but a considerable increase in use during the first year following the death. The use of antipsychotics was much rarer than antidepressants, and there was a statistically significant difference in use only between bereaved parents due to external causes of death and non-bereaved parents.

**Fig 4:**
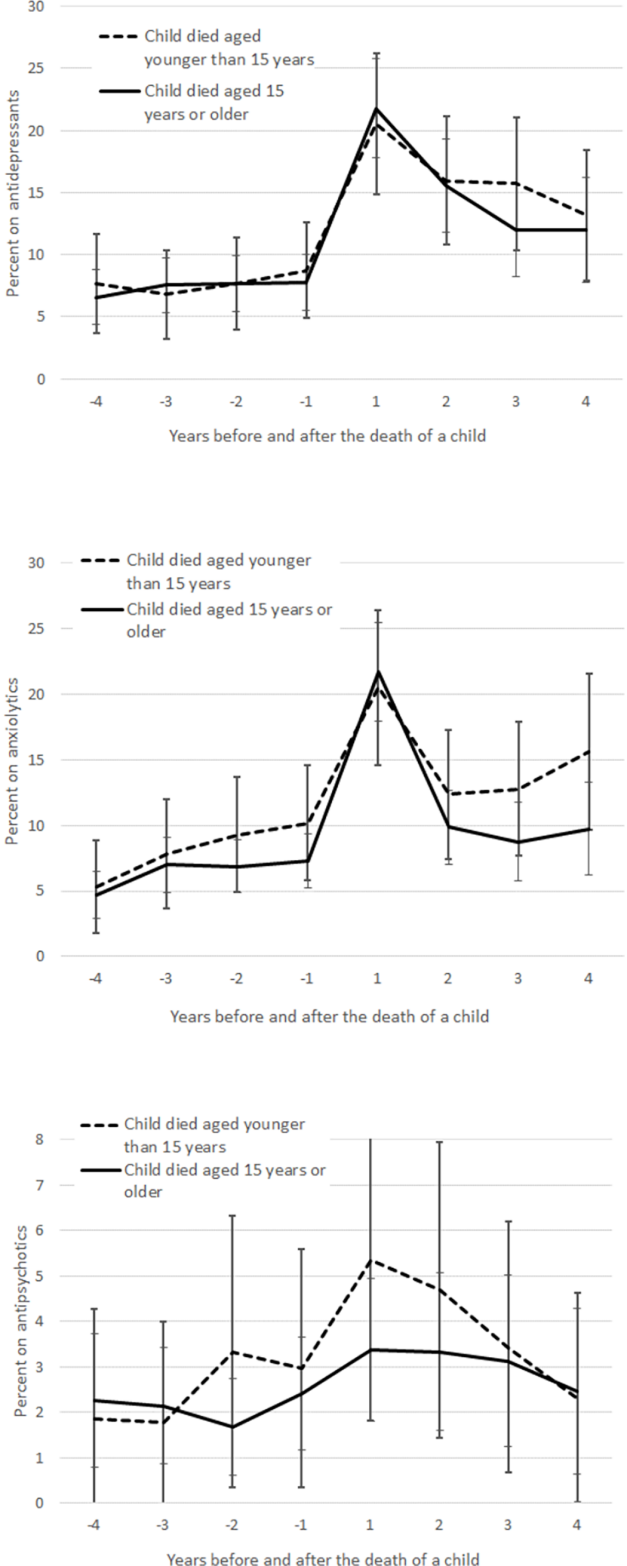
Use of psychotropic medication (percent with 95% Cis) four years before and after the death of a child among parents whose child died by the age of child at death and by medication type (antidepressants, anxiolytics and antipsychotics) and cause of death of a child. Adjusted for parental age and sex, calendar year, number of children under the ages of 7 and 18, health care district, living arrangements, education, occupation based on social class and household income per consumption unit.

For the final analyses, we only included those parents who had lost a child. We were interested in whether the use of psychotropic medication was different among parents whose child died aged younger or older than 15 years (Fig 4). However, young age at death only moderately influenced the use of psychotropic medication in parents, and no systematic patterns were observed.

## Discussion

### Summary of the main results

The aim of this study was to study parents’ use of psychotropic medication before and subsequent to the death of a young child, using population registry data from Finland. We found a marked increase in the use of antidepressants and anxiolytics in mothers and fathers following the death of their child. The highest level of medication use was found around one year after the child’s death, after which use decreased. Regardless, medication use among bereaved parents remained higher than among non-bereaved parents four years after the death of a child or four years after the randomly selected control date, respectively. We also found that the increase in use of psychotropic medication was somewhat more pronounced among bereaved mothers than fathers, and for deaths due to external causes rather than disease-related deaths. Child’s age at death only modestly modified the use of psychotropic medication in parents. Furthermore, psychotropic medication use increased strongly in the one year before death when the child had died from a disease.

### Interpretation

Our findings are likely to reflect the fact that bereavement gives rise to psychiatric symptoms and illnesses ranging from social dysfunction and affective symptoms to more severe psychiatric hospitalization and even suicide [3, 5–14]. Thus, the use of psychotropic medication can be interpreted as a means of coping with underlying psychological and mental health problems following the traumatic event of a child’s death. However, it is also possible that psychotropic medication is used to treat normal grief reactions to painful and traumatic events such as child loss and not merely complicated grief. Our findings also show that the highest level of psychotropic medication use was found in the first year after loss. This is in line with research indicating that psychiatric health problems are most intense in early bereavement [10,23,24]. The finding also corresponds to a recent study showing that elevated rates of psychotropic medication use end within 1 year of the death of an infant child. However, since our results also suggested an increased level in the use of psychotropic medication up to four years after bereavement (and in some cases even prior to bereavement), it is important to acknowledge that child loss could lead to potential long-term harmful effects [27]. A longer-term increase (i.e. after one year) in the use of psychotropic medication was however not found in the aforementioned study on infant death [19]. Thus, other types of bereavement support, such as counselling, should be considered as an alternative to avoid longer-term psychotropic dependency. Future research should also compare patterns of uncomplicated or normal grief to patterns of complicated grief, specifically in relation to the use of psychotropic medication, so as to assess the overall benefits and harms of pharmacological treatments. In particular, clinicians must question whether or not uncomplicated grief is a life situation which can be effectively treated with medication [32].

Our findings indicate a larger absolute increase in the use of psychotropic medication after child loss among mothers when compared to fathers. These findings can be interpreted using theories on social relationship quality and attachment between children and parents. Throughout the life course, gender-related norms and role expectations tend to foster greater attachment of children to mothers than to fathers [33,34]. The mother-child relationship is characterized by more shared values and attitudes, greater affective closeness and higher stability, e.g. in cases of parental separation, than the father-child relationship [35]. This may account for the stronger adverse impact of a child’s death on maternal mental health, as well as its consequent increase in use of psychotropic medication. This finding is supported by previous studies which also suggest more detrimental health consequences for mothers than fathers following the death of a child [2,26]. However, the larger increase in women’s use of medication may also be explained by higher overall depression rates and the increased vulnerability and sensitivity of women to stressful events. Child loss can thus have a greater impact on the mental health of women and thereby contribute to a higher increase in the use of psychotropic medication.

The child’s cause of death can have significant implications for the intensity of a parent’s bereavement symptoms and consequent medication use, which is why we also studied these consequences by distinguishing between disease-related and external causes of death. In most cases, child deaths from external causes such as accidents and suicides are unexpected and can thus have a stronger and more immediate influence on parental wellbeing. This can cause a more severe and complicated grief process [36] and thereby lead to more frequent and possibly longer-term use of psychotropic medication. On the other end of the spectrum, diseases generally take some time to develop and thus death may be experienced with more preparation. Death from natural causes may be eased through more supportive functions within the health care system both before and after the death. Furthermore, parents of children with chronic diseases may have more time to come to terms with the approaching death. Accordingly, our results suggest differing trends of psychotropic medication use between our disease-related and external cause of death child mortality cohorts.

We also found an increase in psychotropic medication use several years before children’s deaths from a disease, indicating that parents may use psychotropic medication to cope with their child’s fatal disease already before the death. Four years prior to a child’s death from a disease, the level of psychotropic medication use is very similar to that of parents who do not experience child death. This fact and the absence of the anticipatory effect for more sudden and unexpected child deaths due to external causes is strong evidence for the causality of the patterns of psychotropic use that we observe in this study.

### Limitations

There were obvious strengths of this study, including the use of a representative sample of the total Finnish population; a longitudinal follow-up design; and reliable information on prescribed use of psychotropic medication with no loss to follow-up or self-report bias. However, some limitations should be noted. More detailed individual information is required to uncover the actual causal mechanisms behind bereaved parents’ use of psychotropic medication. It would be ideal to access and utilize indicators of parental-child attachment, which could amplify or suppress the risk for adverse outcomes. Stronger parental-child attachment may lead to more mental health problems and consequent use of psychotropic medication in bereaved. Moreover, using pharmaceutical purchases as an indicator of psychotropic medication use could pose some limitations, since purchases do not necessarily reflect the severity of underlying mental health problems and the need for medication.

Regardless, our outcome measure of choice exhibited some pertinent strengths. First, accounting for our context, we must acknowledge the role of Finnish medical professionals in prescribing psychotropic medication to patients. This means that the use of such medication is dependent on a clinical assessment of the patient’s actual need for treatment [37]. Changes in the use of such medication are thus likely to denote changes in mental health that are not only observed by the persons themselves but also have direct implications for the provision of medical care. In addition, psychotropic medication use is a significant predictor of other adverse outcomes such as early retirement due to disability [38]. This may have strong implications on predicting patterns of government aid in other social welfare systems. On a final note, our findings may be indicative of other contexts and patterns of psychological need around stressful life events. Similar changes in the use of psychotropic medication before and after divorce have been found in Finnish register-based data as well as in a self-reported trajectory of psychological distress in the UK [39]. Needless to say, these findings should not be generalized without caution. Further studies on serious illness in children and its effects on the mental health of parents may provide further validation of our study results.

### Conclusions

To our knowledge, few population-based studies beyond ours have examined the use of psychotropic medication in bereaved parents around the death of a child. Given the increase in use of psychotropic medication among bereaved parents both before and after the death of a child, our findings indicate that child death can be associated with significant mental distress. Psychotropic medication may be used as a mean to alleviate these symptoms and avoid more severe outcomes such as suicide or psychiatric disease. However, our findings also call for a critical discussion of whether grief, especially uncomplicated grief, is a life situation that should be treated with medication. The results do provide strong evidence for the general idea that stressful life events have significant adverse health effects.

## Supporting information

S1 Fig Structure of the data by period, one year study cohorts, and year. (TIFF)

S1 Table Distribution of the covariates adjusted for n the models by whether parent lost a child or not. Mothers and fathers combined.
(DOCX)

## Abbrevations

CI: Confidence interval
ATC: Anatomical Therapeutic Chemical classification system
