# Supplementary figures and images for: Does the death of a child influence parental use of psychotropic medication? A follow-up register study from Finland
Source: PLoS One. 2018 May 2;13(5):e0195500. doi: 10.1371/journal.pone.0195500 (PMC5931448; doi:10.1371/journal.pone.0195500)

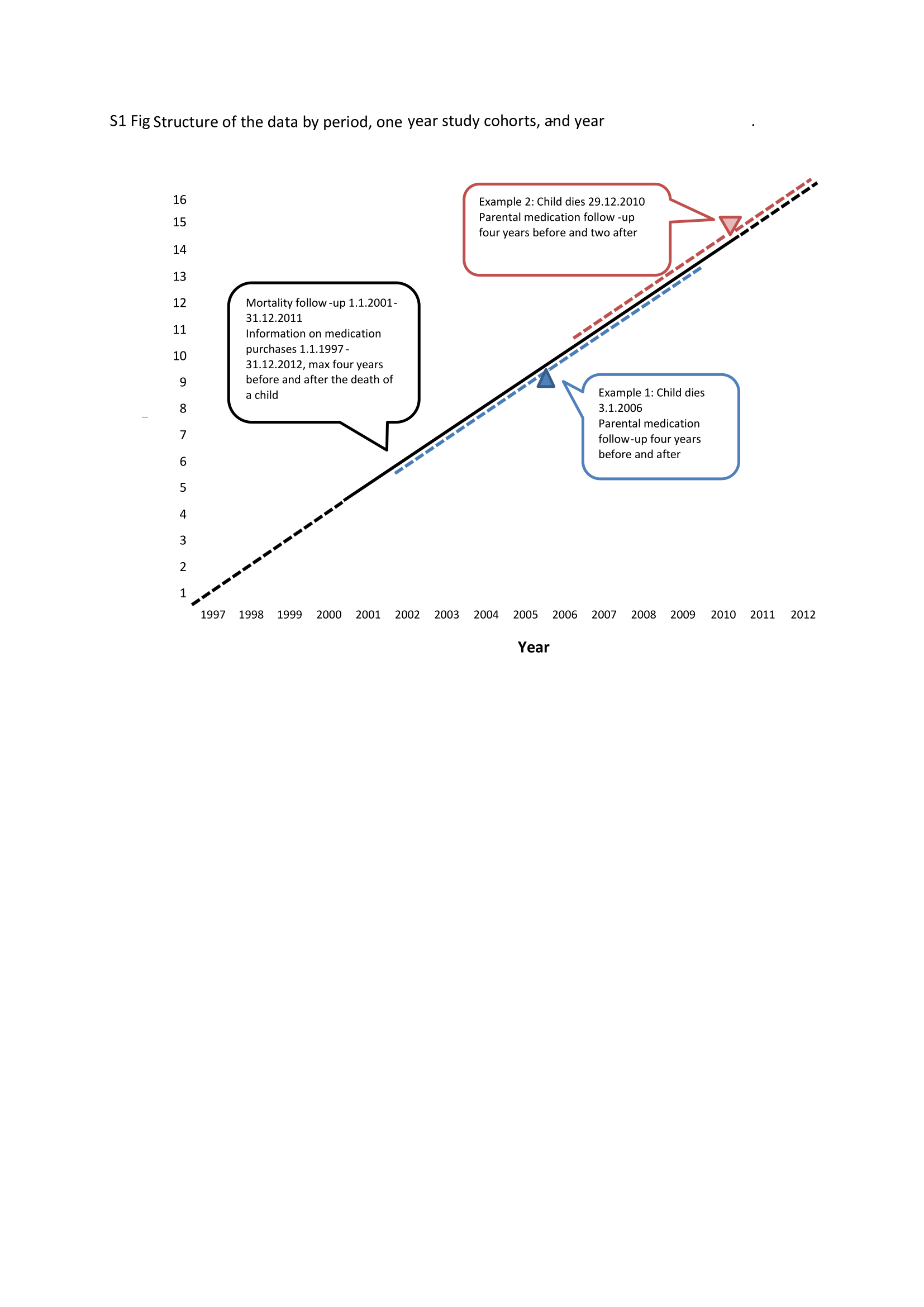

Supplement: S1 Fig — (TIFF) [file pone.0195500.s001.tiff]
