# Supplementary material for: Does the death of a child influence parental use of psychotropic medication? A follow-up register study from Finland
Source: PLoS One. 2018 May 2;13(5):e0195500. doi: 10.1371/journal.pone.0195500 (PMC5931448; doi:10.1371/journal.pone.0195500)
Supplement: S1 Table — Mothers and fathers combined. (DOCX) [file pone.0195500.s002.docx]

S1 Table. Distribution of the covariates adjusted for in the models by whether parent lost a child or not. Mothers and fathers combined.

|  |  | Child died | | Child did not die | |  |  |  | Child died | | Child did not die | |
| --- | --- | --- | --- | --- | --- | --- | --- | --- | --- | --- | --- | --- |
| Parental age in years (mean)^1^ | | 45,9 |  | 42,9 |  |  | Household income € | |  |  |  |  |
|  |  |  |  |  |  |  |  | per consumption unit (mean)^1^ | 23 290 |  | 22 258 |  |
| Living arrangements (%)^1^ | |  |  |  |  |  |  |  |  |  |  |  |
|  | Married or cohabiting without children | 4,5 |  | 4,7 |  |  | Health-care district (%)^1^ | |  |  |  |  |
|  | Married or cohabiting with children | 77,2 |  | 76,9 |  |  |  | Åland | 0,5 |  | 0,4 |  |
|  | Single parent | 10,6 |  | 12,0 |  |  |  | Helsinki and Uusimaa | 27,7 |  | 21,0 |  |
|  | Not part of a family (Biological parent living alone) | 4,4 |  | 1,9 |  |  |  | Southwest Finland | 8,4 |  | 9,2 |  |
|  | Unknown | 3,3 |  | 4,5 |  |  |  | Satakunta | 4,3 |  | 3,7 |  |
|  |  |  |  |  |  |  |  | Tavastia | 3,3 |  | 3,5 |  |
| Number of children in the family aged under 7 years^2^ | |  |  |  |  |  |  | Pirkanmaa | 8,3 |  | 7,3 |  |
|  | 0 or the parent is not part of a family | 81,0 |  | 70,3 |  |  |  | Päijänne-Tavastia | 3,9 |  | 4,2 |  |
|  | 1 | 9,6 |  | 18,6 |  |  |  | Kymenlaakso | 3,4 |  | 3,2 |  |
|  | 2 | 7,6 |  | 9,1 |  |  |  | South Karelia | 2,3 |  | 2,1 |  |
|  | 3+ | 1,7 |  | 2,1 |  |  |  | Southern Savonia | 2,1 |  | 3,2 |  |
|  |  |  |  |  |  |  |  | Eastern Savonia | 1,0 |  | 1,6 |  |
| Number of children in the family aged under 18 years^2^ | |  |  |  |  |  |  | North Karelia | 3,3 |  | 5,3 |  |
|  | 0 or the parent is not part of a family | 34,1 |  | 22,3 |  |  |  | Northern Savonia | 4,7 |  | 4,7 |  |
|  | 1 | 23,8 |  | 27,6 |  |  |  | Central Finland | 5,1 |  | 6,1 |  |
|  | 2 | 23,2 |  | 32,0 |  |  |  | Southern Ostrobotnia | 3,9 |  | 5,8 |  |
|  | 3 | 12,3 |  | 13,2 |  |  |  | Vaasa | 3,1 |  | 1,4 |  |
|  | 4+ | 6,5 |  | 4,9 |  |  |  | Central Ostrobotnia | 1,5 |  | 1,0 |  |
|  |  |  |  |  |  |  |  | Northern Ostrobotnia | 7,5 |  | 10,3 |  |
| Education (%)^2^ | |  |  |  |  |  |  | Kainuu | 1,6 |  | 2,9 |  |
|  | Basic | 20,3 |  | 17,9 |  |  |  | Länsi-Pohja | 1,3 |  | 1,9 |  |
|  | Secondary | 50,7 |  | 44,1 |  |  |  | Lapland | 2,4 |  | 1,1 |  |
|  | Tertiary | 29,0 |  | 38,0 |  |  |  |  |  |  |  |  |
|  |  |  |  |  |  |  |  |  |  |  |  |  |
| Socioeconomic status (%)^1^ | |  |  |  |  |  |  |  |  |  |  |  |
|  | upper non manual | 18,1 |  | 13,6 |  |  |  |  |  |  |  |  |
|  | lower non manual | 30,8 |  | 29,7 |  |  |  |  |  |  |  |  |
|  | manual | 32,4 |  | 38,5 |  |  |  |  |  |  |  |  |
|  | farmer | 3,7 |  | 5,7 |  |  |  |  |  |  |  |  |
|  | entrepreneur | 7,7 |  | 7,2 |  |  |  |  |  |  |  |  |
|  | others (eg. Students) | 7,3 |  | 5,3 |  |  |  |  |  |  |  |  |
|  |  |  |  |  |  |  |  |  |  |  |  |  |

|  | ^1^Time-variant variable. Measured at the beginning of the follow-up |  |  |  |  |  |  |  |  |  |  |
| --- | --- | --- | --- | --- | --- | --- | --- | --- | --- | --- | --- |
|  | ^2^Time-invariant variable. Measured at the turn of the year preceding the date of the death a child / reference date | | | | | | |  |  |  |  |
